# Notes

**Published:** 1898-07-23

**Authors:** 


### Notes.

Medical heroes so rarely obtain recognition that Sir Walter Foster is to be congratulated on bringing before the House of Commons the heroic action of Surgeon-Lieutenant V. Hugo during the Indian Frontier War, who remained under close fire for three hours holding the cut artery of a wounded fellow officer, and finally carried the unconscious man to a place of safety without relaxing his hold on the artery.

The Royal Commission on Sewage Disposal has decided to undertake a considerable amount of bacteriological and chemical work in connection with the matters submitted to it. No further evidence, therefore, will be taken until September. There is no doubt that the whole problem is overrun with patents and private claims, and it will be a great advantage that many of the statements made in regard to the various systems should be thoroughly tested by an independent authority.

The Public Control Committee of the London County Council, having carefully considered the question of the use of water-gas, have come to the conclusion that considerable danger arises from the introduction of water-gas; that non-carburetted and non-odorised water-gas should not be allowed to be used under any conditions, since it is devoid of smell which would give warning of any escape of the gas; that 25 per cent. should be the maximum amount of water-gas allowed to be introduced in the enrichment of coal-gas; and that when it is proposed to supply poisonous enriched gas to the interior of buildings, a proper inspection of the service-pipes should be made by a responsible officer, appointed by the local or other suitable authority, at the expense of the gas company.

The Council of the British Medical Association has resolved, as a memorial of the late Mr. Ernest Hart, to found a scholarship, to be called the "Ernest Hart Memorial Scholarship for Preventive Medicine." The scholarship will be of the annual value of £200, and will be tenable for two years. Both the act and the title are peculiarly appropriate. Nothing could be more proper than that the British Medical Association should establish a permanent memorial of the great services rendered to it in many ways, and during many years, by Mr. Ernest Hart, and no title for such a memorial could be more fitting than one which shall remind those who may perhaps, in the future, associate his name chiefly with journalism, that during a large portion of his life his main interests were bound up in the prevention of disease.

In the House on Commons on Tuesday, on the Vaccination Bill, as amended by the Standing Committee, coming up for consideration, Sir W. Foster moved a new clause, exempting a parent from prosecution if he made a statutory declaration that he entertained conscientious objections to vaccination. In this he received the support of Dr. Farquharson, who believed that if the amendment could be accepted "the game of the anti-vaccinator would be up," although how he arrived at this conclusion was not made very clear. In the end Mr. Balfour came in with a notable suggestion, or compromise if one prefers the term, to the effect that it should be regarded as an offence if any parent failed to show that it was from a conscientious objection to the operation that he failed to have his child vaccinated. He thought that by this means it would be possible to discriminate between the father who was an honest, genuine objector, and the careless, irresponsible parent, whose only object was that of saving himself trouble. If the House was prepared to accept that view, he would make the Bill experimental for a period of five years, and would bring up a new clause that would provide that no parent should be liable for any penalty if he satisfied the Court that he conscientiously believed that vaccination was prejudicial to the health of his child, and that failure to have a child so vaccinated was due solely to such belief on his part. So here the matter stands.

